# Examining Social Influence on Participation and Outcomes among a Network of Behavioral Weight-Loss Intervention Enrollees

**DOI:** 10.1155/2013/480630

**Published:** 2013-06-06

**Authors:** T. L. Carson, K. E. Eddings, R. A. Krukowski, S. J. Love, J. R. Harvey-Berino, D. S. West

**Affiliations:** ^1^Division of Preventive Medicine, School of Medicine, University of Alabama at Birmingham, Birmingham, AL 35294, USA; ^2^Department of Health Behavior and Health Education, Fay W. Boozman College of Public Health, University of Arkansas for Medical Sciences, Little Rock, AR 72205, USA; ^3^Department of Preventive Medicine, University of Tennessee Health Science Center, Memphis, TN 38163, USA; ^4^Department of Nutrition and Food Sciences, University of Vermont, Burlington, VT 05405, USA

## Abstract

Research suggests that social networks, social support, and social influence are associated with weight trajectories among treatment- and non-treatment-seeking individuals. This study examined the impact of having a social contact who participated in the same group behavioral weight-control intervention in the absence of specific social support training on women engaged in a weight-loss program. Participants (*n* = 92; 100% female; 54% black; mean age: 46 ± 10 years; mean BMI: 38 ± 6) were grouped based upon whether or not they reported a social contact enrolled previously/concurrently in our behavioral weight-control studies. Primary outcomes were 6-month weight change and treatment adherence (session attendance and self-monitoring). Half of the participants (53%) indicated that they had a social contact; black women were more likely to report a social contact than white women (67.3% versus 39.5%; *P* < 0.01). Among participants with a social contact, 67% reported at least one contact as instrumental in the decision to enroll in the program. Those with a contact lost more weight (5.9 versus 3.7 kg; *P* = 0.04), attended more group sessions (74% versus 54%; *P* < 0.01), and submitted more self-monitoring journals (69% versus 54%; *P* = 0.01) than those without a contact. Participants' weight change was inversely associated with social contacts' weight change (*P* = 0.04). There was no association between participant and contact's group attendance or self-monitoring. Social networks may be a promising vehicle for recruiting and engaging women in a behavioral weight-loss program, particularly black women. The role of a natural social contact deserves further investigation.

## 1. Introduction 

Social networks, which refer to the web of social relationships that surround individuals, have been associated with the spread of health behavior change [1–3]. Social networks function to provide social capital, social influence, companionship, and social support [3] and thus may foster the contagion of healthy behaviors. When focusing on obesity, *social support* defined by Glanz et al. as “aid and assistance exchanged through social relationships and interpersonal transactions” and *social influence*, the “process by which others' thoughts and actions are changed by actions of others,” are commonly examined as functions of social networks that may be useful for improving weight-loss outcomes [3]. 

Social networks, social support, and social influence have all been associated with weight loss and/or weight status in treatment- and non-treatment-seeking populations [4–10]. Links between weight change and interpersonal relationships have been demonstrated in both observational and behavioral intervention trials. Christakis and Fowler [4] reported on a large cohort followed over 32 years and found that an individual's chances of becoming obese over this extended interval increased if he or she had a friend, sibling, or spouse who became obese during that period. Researchers have also found that untreated spouses of participants receiving an intensive behavioral weight-loss intervention in a large, multicenter randomized controlled trial lost more weight than the spouses of participants in the control group which did not receive the weight loss program; furthermore, weight loss in the spouse was positively associated with participant weight loss [7]. Thus, there is a reason to suspect that one's natural social contacts may play a vital role in his or her weight loss or weight gain.

The role of social influence is a core pathway for behavior change and sustained behavior maintenance within the social cognitive theory, which is the conceptual foundation of most current behavioral interventions [3, 11]. One key concept of the social cognitive theory is *observational learning or modeling, *which is “behavioral acquisition that occurs by watching the actions and outcomes of others' behavior” [3]. Therefore, supported by the social influence construct, we posit that positive weight-related behavior change observed in a close associate can provide a credible role model and promote positive weight-related behavior changes in the participant. Thus, even in the absence of a formal role as a supporter, a social contact who is engaged in weight-management behaviors may influence an individual to become more engaged in weight-loss efforts. 

Previous research studying the association of weight loss and treatment adherence of participants in a behavioral weight-loss program has focused on individuals who were enrolled together for the purpose of being support partners [5–7, 12]. To date, there have been no studies that have examined the direct association of weight loss and treatment adherence between study participants and that of their social contacts who were also previous or current program participants. The purpose of this study was to examine whether participants in a behavioral weight loss program, from this point forward referred to as the *index participants*, who indicated that they knew another program participant would achieve greater weight loss than individuals who did not know another program participant. Given the well-documented disparities in weight losses of black women compared to white women [13] and the suggestion that social support may be particularly relevant among black women [14], we also examined outcomes by race. Additional subgroup analyses were conducted comparing outcomes of individuals who knew a previous participant versus those who knew a concurrent participant in order to explore whether temporality played a role in observed outcomes. Finally, we analyzed whether weight loss, attendance, and self-monitoring of the index participant were associated with those same outcomes of the person whom they identified as a social contact.

## 2. Methods

### 2.1. Participants

Data for this ancillary study were collected for 92 women (index participants) from the Arkansas site of a multicenter (Burlington, VT and Little Rock, AR, USA) randomized controlled trial of group behavioral weight control delivered online. For the overall trial, overweight and obese volunteers were recruited by emailed notices, advertisements placed around the community, and word of mouth. To be eligible, individuals were required to be over 18 years old, have a BMI between 25 and 50, be generally healthy, able to walk for exercise, have access to a computer with an Internet connection, and be willing to accept randomization to an online group-based behavioral weight-control program with and without the addition of individual online motivational interviewing counseling. Individuals were excluded if they reported a history of major medical problems for which weight loss was contraindicated, were currently engaged in other weight-loss treatment, had a history of gastric bypass surgery, reported recent significant weight loss, or lived at great distance from one of the clinical centers. Pregnancy within the previous 6 months and current breastfeeding were also exclusion criteria, as was a plan to move from the area during the study period. All randomized participants received the same behavioral weight-control program at no charge. The behavioral weight-control program was an evidence-based program that was delivered to all participants over the internet in the format of weekly chat sessions for 6 months. It included behavioral strategies shown to promote change such as self-monitoring, goal setting, stimulus control, nutrition education, and cognitive restructuring [15, 16]. In addition to the standard behavioral weight-control program, the active treatment group also received motivational interviewing sessions via online chat in order to test whether including this additional component improved weight-loss outcomes. 

The trial was approved by the Committee on Human Research in the Behavioral Sciences at the University of Vermont and the Institutional Review Board at the University of Arkansas for the Medical Sciences. The current ancillary study examines only participants enrolled at the Arkansas site who provided social contact data. Three consecutive recruitment waves were included in the present analysis and were enrolled between 2010 and 2011.

### 2.2. Measures

#### 2.2.1. Body Weight and Height

Weight was measured in light, indoor street clothing, without shoes, on a calibrated digital scale. Weight was measured at baseline and 6 months. Height was measured using a wall-mounted stadiometer (Seca, Hanover, MD, USA) at baseline. BMI was calculated as weight (kg)/height (m^2^). Weight change at 6 months was calculated as difference from baseline.

#### 2.2.2. Social Contact

Index participants were asked to indicate whether they knew any past (previous) or current (concurrent) participants from one of our behavioral weight-control research interventions*. This included participants in similar behavioral weight-control studies delivered by our research team from 2003 to the present study.* If they responded yes, they were further probed to elicit the social contact's name, relationship (“family,” “friend,” “coworker,” or “other” such as friend of a friend, casual acquaintance, or fellow church member), whether or not the social contact's participation was instrumental in their decision to participate in the study (yes or no), and how closely they rated their relationship (very close, somewhat close, or not very close). 

#### 2.2.3. Treatment Engagement

Group attendance and self-monitoring are important measures of treatment engagement and are both strongly and positively associated with magnitude of weight loss in behavioral interventions [17–19]. Thus, both engagement measures were examined to determine whether there were associations between the degree of treatment engagement demonstrated by an index participant and her social contact(s). Group attendance was defined as a participant attending an onsite group session or as logging in for weekly group chat sessions (attending or chatting at a make-up session was also considered). Self-monitoring was determined based on whether a participant submitted a weekly food journal (paper or electronic) to her group facilitator. In order to allow for comparison of behavior of participants across multiple studies of differing duration, attendance and submission of weekly self-monitoring journals detailing daily food/beverage intake and physical activity were calculated as proportions. Proportion of sessions attended was calculated by dividing the total number of weekly sessions attended by the total number of sessions offered. For the current study, 24 weekly sessions were offered. Similarly, the proportion of total expected self-monitoring journals submitted was calculated to allow for comparisons across studies. A total of 23 journals were possible for the current study. The consistency in treatment expectations across programs with respect to attendance and self- monitoring allows comparison of treatment engagement across different programs. 

### 2.3. Statistical Analysis

Baseline comparisons of those with and without social contacts were completed using independent *t*-tests for continuous variables and chi-square tests for categorical variables. Analysis of covariance (ANCOVA), controlling for baseline weight and race, was used to compare weight change of individuals with and without social contacts. Baseline observation carried forward was used for individuals missing 6-month follow-up weights [20, 21]. Completers-only analyses were also conducted for primary outcomes. Exploratory subgroup analyses were completed for participants with a social contact in which independent *t*-tests were used to compare weight change of participants by race (black versus white) and by participation period of their social contact (previous versus concurrent). 

Associations of weight losses between index participants and their social contacts were examined using generalized linear models. Generalized estimating equations (GEE) were used to account for multiple observations of the same individual [22], that is, one index participant with multiple social contacts, one social contact for multiple index participants, or an individual who is both an index participant and a social contact. GEE models were controlled for index participant's race, baseline weight, and the social contact's baseline weight. Similar generalized linear models were used to examine associations between weight-related behaviors (e.g., group attendance and self-monitoring) of index participants and their contacts. Model covariates were selected based upon associations identified in univariate analyses or the literature-based support for inclusion. 

## 3. Results

**Figure 1:**
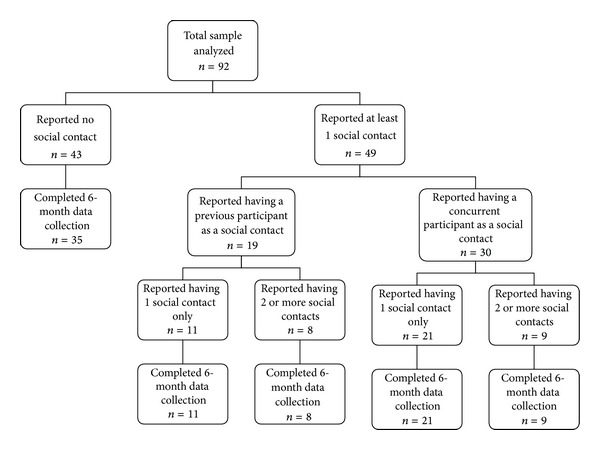
Flow diagram of index participants' reporting of a social contact who is a previous or concurrent study participant.

Index participants were 92 overweight and obese females (age 45.8 ± 9.7 years) with a mean baseline BMI of 38.1 kg/m^2^. Fifty-four percent of index participants were black. Over half (53%) of index participants named at least 1 social contact who was also a member of one of our behavioral weight-control studies (previous or concurrent) (Table 1). A small proportion of total index participants indicated having 2 contacts (11%) or 3 contacts (8%) (Figure 1). A greater proportion of index participants with a social contact were black compared to those with no social contact (67.3% versus 39.5%; *P* < 0.01). Unadjusted analyses indicated that there was no difference in age of index participants with and without social contacts; however, participants with social contacts weighed more at baseline than those without contacts (*P* = 0.02) (Table 1). Since black participants were more likely to have a social contact and were heavier at baseline (105.5 versus 97.2 kg; *P* = 0.03) than whites, we adjusted for race in all subsequent analyses. After adjusting for race, the difference in baseline weight between those with a contact and those without was no longer significant, although it still trended in the direction of greater weight among those with a social contact (*P* = 0.07). 

There were 55 unique individuals identified as a social contact, indicating that the same individual was listed as a contact for multiple index participants; a total of 73 distinct index/contact relationships were reported.  Table 2 describes the sociodemographic characteristics of the 55 unique social contacts. Approximately one-third of social contacts were previous program participants, that is, participants in a completed study in which our research group delivered a similar behavioral weight-control intervention (Table 2). The remaining social contacts (65%) were concurrent participants in the present study. The nature of the social contact for index participants with a single social contact (*n* = 32) is described in Table 3. Most contacts were identified as a friend or coworker (86%). Over two-thirds (67%) were reported as having played an instrumental role in an index participant's decision to enroll. 

Our primary analysis compared index participants with a social contact (previous and/or concurrent) who had also participated in a structured weight-loss program to those without a social contact. After adjusting for baseline weight and race, individuals with a social contact lost more weight (5.9 versus 3.7 kg; *P* = 0.04) than participants who did not identify a social contact. Participants with a social contact also had better adherence. Specifically, those with a contact attended more group sessions (74% versus 54%; *P* < 0.01), submitted more journals (69% versus 54%; *P* = 0.01), and had a greater proportion of completed 6-month data collection compared to participants with no social contact (100% versus 81%; *P* = 0.01). Unadjusted analyses also indicated that the behavioral adherence measures, that is, attendance and journaling, were positively associated with weight loss (*r* = 0.54, *P* < 0.001; *r* = 0.62, *P* < 0.001, resp.). Therefore, we conducted subsequent mediation analyses, which revealed that the relationship between whether or not an index participant had a social contact and her weight-loss outcome was mediated by these indicators of behavioral adherence. Although there was relatively high retention in the study overall (91% provided 6-month data), dropout occurred in one group exclusively (*N* = 8), all of whom were from among participants with no social contact. Thus, completers-only analyses were conducted to explore potential biases introduced by employing baseline carried forward imputation in the primary analyses. Completers-only analyses revealed similar trends to the intent-to-treat analysis (5.9 among those with a social contact versus 4.5 kg among those without a social contact), though the 1.4 kg difference in weight losses between groups was no longer statistically significant (*P* = 0.18). Results from additional analysis across all 3 groups—no contact, previous contact, or concurrent contact—are displayed in Table 1. 

### 3.1. Subgroup Analysis of Participants with Social Contact

Subgroup analyses examined the sociodemographic characteristics of individuals with social contacts to determine whether distinct patterns emerged that identified which participants in particular appeared most likely to benefit from a social contact. Among black and white women with a social contact, there were no statistically significant differences in the weight losses (5.4 versus 7.1 kg, resp.; *P* = 0.29) or percent of baseline body weight lost (5.1% versus 7.1%, resp.; *P* = 0.21). Additionally, there were no differences in group attendance (74% versus 74%; *P* = 0.99) or self-monitoring journals submitted (65% versus 76%; *P* = 0.26) by blacks and whites with a social contact, respectively. Thus, black and white women both appeared to benefit in weight loss outcomes and treatment engagement with a social contact. Comparisons of women who reported a previous-participant social contact versus those with a concurrent-participant social contact did not reveal significant differences in weight loss (5.2 versus 6.4 kg, *P* = 0.42; previous versus concurrent, resp.) achieved by the women. Further, adherence of participants did not differ based on whether the social contact was currently or previously enrolled. Specifically, there was no difference in group attendance (75% versus 73%, *P* = 0.80; previous versus concurrent, resp.) or journals submitted (66% versus 70%, *P* = 0.66; previous versus concurrent, resp.). Therefore, it would appear that social contacts who had engaged in the treatment program some time previously were as effective in conferring benefit as were those currently enrolled. There was no difference in weight losses when comparing participants who reported their contact as instrumental to their participation to those who were not instrumental (*P* = 0.30). Finally, the participant's rating of the closeness of her relationship with her social contact was not associated with weight loss (*P* = 0.50), journaling (*P* = 0.60), or group attendance (*P* = 0.79). 

### 3.2. Association of Weight-Loss Outcomes and Treatment Adherence between Index Participant and Social Contact

The social contact's weight change was a significant predictor of the index participant's weight change when controlling for the index participant's race, baseline weight, and the social contact's baseline weight (*β* = −0.20, *P* = 0.03), such that, as a social contact's weight loss increased, the index participant's weight loss decreased. There was no association between index participant's and social contact's group attendance (*P* = 0.38) or weekly journals submitted (*P* = 0.66). When stratified by social contact's participation temporality (previous versus concurrent), the social contact's weight change was a significant predictor (*β* = −0.44, *P* = 0.02) of the index participant's weight change with adjustment for the index participant's race, baseline weight, and the social contact's baseline weight for those whom the social contact was a previous participant. This relationship was not observed among those for whom the social contact was a concurrent participant (*β* = −0.20; *P* = 0.10). 

## 4. Discussion

The findings of this study suggest that natural social networks can be a promising vehicle to promote recruitment for and engagement of participants in a behavioral weight loss program. This was particularly true for black women. Over half of our study participants reported having a social contact who also participated in a similar weight-loss program and, of those, the majority said that their social contact's participation played a key role in their decision to participate in this program. Index participants with a previous or concurrent participant as a social contact also attended more group sessions, submitted more weekly food journals, and lost more weight than those with no social contact. Mediation analyses suggested that the relationship between presence or absence of a social contact and weight loss was mediated by behavioral adherence measures. Thus, behavioral adherence explained the relationship between the presence or absence of social contact and weight loss, which highlights the need for a better understanding of how having a social contact who has participated may lead to greater engagement in a weight-loss program for another participant. 

Our study is consistent with previous studies that have demonstrated that healthy choices can be spread through social ties [2, 4, 9]. For example, Leahey and colleagues have shown that having more social contacts trying to lose weight is associated with greater weight-loss intentions in young adults [9]. Similarly, individuals in the current study indicated that their social contacts' participation played a key role in their decision to join the current study, further demonstrating that having a social contact who has or is trying to lose weight can influence an individual's decision to also engage in weight-loss efforts. We also found that participants with a social contact who participated in a similar weight-loss program lost more weight than participants with no social contact from the program. Participants with a social contact also appeared to be more engaged than those without a social contact as evidenced by higher group attendance, more self-monitoring journals submitted, and more complete data collection at 6-month followup. Similar relationships were seen in an earlier social support study in which participants recruited with friends and treated with a social support intervention had better attendance and less dropout than those recruited alone [8]. The findings of the current study provide additional support for the utility of social networks for recruitment and retention of women in behavioral weight-loss studies. Though it is not fully clear why participants with a social contact engaged in treatment at a higher level than those with no social contact, it is of vital importance to understand why participants with a social contact engaged at a high level since mediation analyses revealed that engagement is what ultimately explained the observed relationships. One hypothesis for greater engagement by women with a social contact may be that the common experience of participating in a behavioral weight loss study with a member of one's social network naturally fostered ongoing social support and accountability even in the absence of specific training in social support strategies. This elevated social support may have contributed to better engagement in the program. The study did not collect social support measures and therefore this hypothesis cannot be confirmed.

Despite the apparent benefit of a social contact in engaging a study participant, index participants and their social contacts in this study did not display similar behaviors. Specifically, we did not find an association between group attendance or self-monitoring of participants and that of their social contacts. These findings were inconsistent with previous social support studies such as multiple reports by Gorin et al. [6, 7] in which there were positive associations between weight losses of participants and their social contacts. The discrepancy between the findings of the Gorin et al. studies and the current report may be explained by differences in study designs. For example, in one study, Gorin and colleagues [6] encouraged study participants to enroll social contacts with the specific intent of being support partners to participants as a part of a larger randomized trial. Given that participants were recruited and enrolled together in a study focused on fostering social support, we might expect that behaviors among these individuals would be more similar than 2 people enrolled in a weight-loss study who simply report that they know one another. We also observed an unexpected inverse relationship between weight change of index participants and their social contacts' such that, as social contact's weight loss increased, the index participant's weight loss decreased. This finding was very counterintuitive and, if true, could have substantial theoretical implications for current popular opinion about the utility of social networks for the promotion of weight loss. While theories such as social comparison processes (upward and downward comparisons) have been considered here as a possible explanation, this is still controversial given the complexity of social comparison processes. Given that this study is exploratory in nature, we are limited in what we can conclude from such a finding, which may, in fact, be an artifact of the data. Instead, this unexpected finding should be further investigated to determine if this association is true in order to provide a better understanding of potential positive and negative influences of social contacts who have achieved various levels of weight loss. 

Our findings support the notion that healthier behaviors can be promoted through a social network even in the absence of formal training or instruction on providing support to a social contact. Not only have our study participants, past and present, engaged in a behavioral weight-loss intervention to attempt weight loss for themselves, but their participation also influenced the decision of others to participate in a similar program. Even though there was no evidence that participants and their social contacts behaved similarly or achieved similar weight losses, the finding that a social contact influenced a woman's decision to participate in weight-control efforts has implications for recruitment and engagement in intervention research. Further, participants with a social contact performed better than those with no social contact. Based on these findings, we can conclude that the role of a social contact in weight-loss efforts deserves further investigation. This is particularly noteworthy given the predominance of black women in this study who reported an influential social contact. Black women are at high risk for obesity and related comorbidities [23, 24] and often are wary of enrolling in research trials [25, 26]. Social facilitation may offer particular benefit to enrolling black women in such trials [27]. We are encouraged by the average 5% of baseline body weight lost by black women with a social contact in this study; weight loss of this magnitude is comparable to some of the best average weight losses reported to date in a behavioral weight-control intervention among black women. For example, the diabetes prevention program lifestyle intervention arm reported an average of 4.9% weight loss among black women at 6 months [13], and that intervention included meal replacements and significantly greater financial resources to facilitate weight loss than available in our intervention [28]. Our findings suggest that, in addition to facilitating recruitment into studies, having a social contact may be conducive to facilitating weight-loss among black women in behavioral weight loss studies. 

The strengths of this study include the racial diversity of the sample, which increases generalizability to high-risk populations, and the ability to explore whether the temporality of a social contact's participation (i.e., past or concurrent participation) had a differential effect on outcomes. Examining the social influence of both past and current social contacts within the same study extends the current social influence literature which primarily focuses on either concurrent weight-loss study of social contacts [6–8, 12] or looks at temporal weight-change trajectories among members of a social network who are not explicitly engaged in a weight-control intervention [4]. This study also has limitations. By nature of the study design, we cannot infer causality. Thus, our findings are limited to establishing associations for further exploration. Additionally, we do not have data from previous participants on several relevant factors. Specifically, we do not have any measures on the current weight status of social contacts that were previously enrolled in the weight-loss intervention. Thus, we cannot determine whether a previous social contact is serving as a real-time model or a historic model for current participants. Also, we did not collect data on the nature of the relationship (e.g., type of relationship, closeness of relationship, influence on participation) from the social contact. Therefore, we were unable to explore relationship bi-directionality, which has been demonstrated as relevant in other studies of social networks [4]. We are limited to examining the perceptions of the index participant, which may not be shared by the social contact, perhaps explaining why the closeness of the relationship in the current analyses was not a relevant factor to the weight-loss outcomes. Further, we did not examine whether individuals received direct social support from other individuals outside of our weight-loss programs, nor did we obtain measures of perceived social support to quantify the relationships. Finally, our sample size was fairly small for subgroup analyses and thus we may not have had adequate power to detect meaningful differences in the subgroups.

In summary, our study shows that natural social influence may be a useful tool to encourage individuals to engage in a behavioral weight-control program. Thus, individuals who decide to attempt weight loss may lead to other members of their social network engaging in weight-loss efforts. Additional research is needed to understand how to effectively leverage social influences to promote positive weight-related behaviors among members of a shared social network. 

## Figures and Tables

**Table 1:** Characteristics of study sample (*n* = 92).

	Total samples (*n* = 92)	Report no social contact (*n* = 43)	Report previous social contact (*n* = 19)	Report current social contact (*n* = 30)	*P* value^a^
Baseline sociodemographic factors					
Black (%)	54.3	39.5	68.4	66.7	0.03
Age (yrs)	45.8 ± 9.7	47.4 ± 10.5	45.2 ± 10.4	43.7 ± 7.6	0.26
Weight (kg)	101.7 ± 17.7	97.1 ± 16.6^e^	101.9 ± 19.2^e,f^	108.1 ± 16.9^f^	0.03
BMI (kg/m^2^)	38.1 ± 6.0	36.8 ± 6.1^e^	37.7 ± 5.3^e,f^	40.5 ± 5.8^f^	0.03
Weight loss at 6 months^b^					
Total weight loss (kg)	4.9 ± 5.5	3.7 ± 5.7	5.2 ± 4.6	6.4 ± 5.5	0.11
% of weight loss	4.9	3.9	5.3	6.1	0.25
Behavioral adherence measures^b^					
% of group sessions attended (out of 24)	64.5	53.6^e^	74.8^f^	73.1^f^	<0.01
% of journals submitted (out of 23)	62.1	54.3	66.3	70.1	0.12

Mean ± SD; ^a^ *χ* ^2^ test for categorical variables, independent *t*-test, or analysis of variance for continuous variables; ^b^controlled for baseline weight and race; ^e,f^means with common superscripts across columns are not significantly different based on Bonferroni post hoc analyses (*P* ≤ 0.05).

**Table 2:** Sociodemographic characteristics of individual social contacts (*n* = 55).

	Total (*n* = 55)	Previous participant (*n* = 19)	Concurrent participant (*n* = 36)	*P* value^a^
Baseline sociodemographic factors				
Black (%)	72.7	78.9	69.4	0.34
Female (%)	96.4	94.7	97.2	0.58
Age (yrs)	44.3 ± 9.5	44.2 ± 10.7	44.3 ± 9.0	0.10
Weight (kg)	104.1 ± 16.1	106.3 ± 18.9	104.1 ± 16.1	0.64
BMI (kg/m^2^)	39.0 ± 5.3	39.4 ± 5.4	38.8 ± 5.3	0.69
Weight loss at 6 months				
Total weight loss (kg)	7.7 ± 5.9	8.7 ± 4.3	7.2 ± 6.6	0.35
% of weight loss	7.7	8.7	7.1	0.33
Behavioral adherence measures				
% of group sessions attended	78.2	82.3	75.9	0.32
% of journals submitted	75.7	84.3	71.1	0.08

^a^ *χ* ^2^ test for categorical variables, independent *t*-test for continuous variables.

**Table 3:** Nature of contact for those with a single social contact (*n* = 32).

	Total (*n* = 32)	Previous (*n* = 11)	Concurrent (*n* = 21)	*P* value^a^
Relationship (%)^b^				
Family	6.9	22.2	0.0	
Friend	48.3	77.8	35.0	<0.01
Coworker	37.9	0.0	55.0	
Other	6.9	0.0	10.0	
How close (%)^c^				
Very close	50.0	80.0	35.0	0.10
Somewhat close	40.0	10.0	55.0	
Not very close	10.0	10.0	10.0	
Race concordant (% yes)	87.5	90.9	85.7	0.57
Instrumental to participation (% yes)^b^	66.7	70.0	65.0	0.86

^a^ *χ* ^2^ test for categorical variables ^b^missing for 3; ^c^missing for 2.
